# Breastfeeding needs of mothers of preterm infants in China: a qualitative study informed by the behaviour change wheel

**DOI:** 10.1186/s13006-023-00587-9

**Published:** 2023-09-01

**Authors:** Yaqi Yu, Qianru Liu, Xiaoju Xiong, Ying Luo, Wen Xie, Wenshuai Song, Maoling Fu, Qiaoyue Yang, Genzhen Yu

**Affiliations:** 1 Nursing Department, Tongji Hospital, Tongji Medical College, Huazhong University of Science and Technology, No. 1095, Jiefang Avenue, Wuhan, China; 2 School of Nursing, Tongji Medical College, Huazhong University of Science and Technology, No. 13, Aviation Road, Wuhan, China; 3 Nursing Department, Union Hospital, Tongji Medical College, Huazhong University of Science and Technology, No. 1277, Jiefang Avenue, Wuhan, China

**Keywords:** Breastfeeding, Mothers, Preterm Infant, Barriers and facilitators, Behaviour change wheel, Neonatal intensive care unit, Separation, Qualitative study

## Abstract

**Background:**

Although breastfeeding is strongly recommended, the breastfeeding rate of preterm infants in China remains significantly low. In addition to the global structural challenges to breastfeeding and the physiological immaturity of preterm infants, Chinese mothers of preterm infants face unique challenges of maternal-infant separation after birth. Moreover, little is known about Chinese mothers’ specific needs in coping with the difficulties posed by these challenges. This study utilized the Behaviour Change Wheel to investigate the breastfeeding needs of Chinese preterm mothers that may facilitate its practice in the future.

**Method:**

A qualitative descriptive design was implemented in Wuhan in 2022. Based on purposeful sampling, 13 preterm mothers were recruited from a NICU in a Grade III Class A hospital in Wuhan, China. Face-to-face semi-structured interviews were conducted to collect data using the interview guide developed by the Theoretical Domains Framework. Theoretical Thematic Analysis was used to review the data in 6 steps to identify themes.

**Results:**

Five major themes emerged: (1) capability: ability to interpret infants’ cues and identify problems, and need for breastfeeding knowledge and skills training; (2) physical opportunity: cleanliness and quietness in household environment, private lactation spaces and breastfeeding tools in workplaces and hospitals; (3) social opportunity: family support, peer support, and authoritative support from healthcare providers; (4) reflective motivation: information on health impacts of breastfeeding; (5) automatic motivation: maternal-infant bonding, free of aversive stimulus.

**Conclusion:**

Preterm mothers’ needs to enable breastfeeding were diverse, including increasing their capability, physical and social opportunities, and reflective and automatic motivation. People, resources and environments associated with these needs should be engaged together to stablish a conducive structural environment for breastfeeding. The policy change for “zero separation” and implementation of kangaroo care should also be implemented in Chinese neonatal intensive care units. Future studies are needed to design effective interventions according to mothers’ specific needs.

**Supplementary Information:**

The online version contains supplementary material available at 10.1186/s13006-023-00587-9.

## Introduction

Breastfeeding has been recognized as the gold standard for all infants, especially preterm ones [1], who typically suffer more health problems due to immaturity. Breastfeeding and breast milk has various positive effects on their health status, including decreasing morbidity of respiratory infection [2], retinopathy of prematurity (ROP), necrotizing enterocolitis (NEC) [3] and early childhood obesity [4], and promoting the nervous system development [5], among others. Additionally, published evidence demonstrates that breastfeeding women have a decreased risk of ovarian and breast carcinoma, lower blood pressure, and greater significant postpartum weight loss [6]. Consequently, breastfeeding plays an especially vital role in the health of preterm infants and their mothers.

Despite the numerous health benefits of breastfeeding, global breastfeeding rates for preterm infants remain low (e.g., Sweden, 45% in 2013; UK’s Northern region, 35.7% in 2018) [7, 8] and are lower to those of their full-term counterparts [9]. Mothers worldwide face several structural challenges in breastfeeding, including gender inequities, harmful sociocultural infant-feeding norms, corporate marketing practices, and poor support for breastfeeding in key settings such as households, workplaces, and healthcare systems [10]. All of these factors significantly impact the practice of breastfeeding. Additionally, for mothers of preterm infants, prematurity poses further challenges at the individual level. Dysphagia, weak sucking and sleepiness in preterm infants lead to higher rate of unsuccessful breastfeeding [11]. The fatigue, frustration, and reduced self-efficacy associated with breastfeeding challenges associated with prematurity further contribute to cessation of breastfeeding [12].

In China, the exclusive breastfeeding rates of preterm infants at 1 month, 3 months, and 6 months after discharge were found to be 19.0%, 17.2%, and 10.4% respectively [3], which was significantly lower compared to the rate of exclusive breastfeeding under 6 months for upper middle-income countries (38.4%) [13]. The structural challenge that most of the Chinese NICU imposed a separation policy on mothers and preterm infants [14] may be the primary cause of low preterm breastfeeding rates in China. In accordance with the latest guidance from the World Health Organization (WHO), it is strongly recommended that preterm infants be exclusively breastfed until 6 months of age, while initiating Kangaroo Mother Care (KMC) as routine care immediately after birth [15]. KMC, an early and continuous method of skin-to-skin contact between the mother and baby, has been associated with decreased all-cause mortality rates, reduced incidence of infection and hypothermia, as well as increased weight gain and breastfeeding in preterm infants [15]. However, these can’t be implemented if infants were separated from their mothers after birth. With China’s fertility policy gradually changing to a policy that officially allows a couple to have three children on August 20, 2021, women of advanced maternal age and the risk of preterm birth may increase [16]. Therefore, how to promote breastfeeding of preterm infant mothers is an urgent problem to be solved.

A diverse range of effective interventions have been implemented globally to make a difference for breastfeeding outcomes [17]. A critical review found that one limitation of intervention strategies for improving breastfeeding is the lack of a sound theoretical framework [18]. According to a previous systematic review, only 3 of the 24 studies on interventions to improve breastfeeding used theory [19]. Among those studies that applied theories, the theory of reasoned action, the theory of planned behavior, and the breastfeeding self-efficacy theory were widely used. These theories focused on creating positive breastfeeding outcomes via modifiable individual factors (e.g., ability, attitude and intention) [20]. However, it’s important to note that breastfeeding a preterm infant is not only a biological process but also a kind of health behavior influenced by a wide range of factors [21]. Individual factors alone may be insufficient, as mothers need environmental and social support as well.

Besides, focusing on needs may contribute to better understanding of behavior change and lead to better intervention outcomes [22]. However, in current promotion practice, healthcare workers lack awareness and attention to maternal needs in breastfeeding [23]. Given the disparities in cultural backgrounds, healthcare policies, economic, and social contexts, Chinese mothers of preterm infants encounter distinctive challenges and may process unique requirements for breastfeeding support.

In order to design effective intervention for breastfeeding of preterm mothers in China, a systematic theory is needed to comprehensively analyze their breastfeeding behavior and their needs when implementing the behavior. The Behaviour Change Wheel (BCW) is a comprehensive framework that can analyze behavior regarding both individual (capability, and motivation) and objective (opportunity) factors. It is a three-layer wheel-shaped model. The hub of the wheel identifies the sources of behavior that can serve as intervention targets, involving three essential components: Capability, Opportunity, and Motivation System (COM-B) [24]. It emphasizes that the target behavior can only occur if an individual possesses the physical (skills) and psychological (knowledge) capability to act, the physical (resources) and social opportunity (cultural) to enable, and automatic (impulses) and reflective (plan) motivation to push forward [24]. The COM-B system can explain how interactions between capability, opportunity, and motivation influence behavior. After influences on behavior are recognized, they can be linked to nine kinds of intervention functions and seven kinds of policy categories, comprising the middle and outer layer of the wheel, respectively [25]. The Theoretical Domains Framework (TDF) is an integrated framework with 14 domains, constituting a more detailed form of the COM-B system [25]. For example, the COM-B system divides capability into physical and psychological capability, whereas the TDF further divides psychological capability into domains, such as knowledge, interpersonal communication, memory, attention, and decision-making competencies, allowing researchers to unravel what may affect target behavior and to identify precisely what needs to be addressed in future interventions.

The BCW has been increasingly applied to breastfeeding recently, such as to develop a motivational interview about breastfeeding peer-support intervention [26], to explore infant breastfeeding peer support provision in North West UK [27], and to investigate breastfeeding experience and intention [28]. While the BCW has not been employed to explore mothers’ breastfeeding needs, it offered a useful analytical framework to help understand breastfeeding behavior from the preterm mothers’ perspective and what support were in needed. An in-depth, theoretically supported research on breastfeeding needs may facilitate effective intervention to enhance preterm mothers’ breastfeeding behaviors and thus increase the breastfeeding rate of preterm infants in China.

Accordingly, this study aims to understand and clarify Chinese preterm mothers’ needs regarding breastfeeding based on the BCW to provide insight into the future design of breastfeeding behavior-enhancing interventions.

## Methods

### Design

A qualitative descriptive design was applied by undertaking individual semi-structured interviews to explore the mothers’ need for breastfeeding. The study adhered to the Consolidated Criteria for Reporting Qualitative Research (COREQ) [29].

### Participants

The preterm infants were treated at a level-III NICU in a Grade III Class A hospital in Wuhan, the provincial capital of Hubei in central China. The recruitment of mothers was based on a purposeful sampling approach with the maximum variety in terms of education, occupation, monthly family income, delivery mode, gestational age, and parity, aiming to ensure the diversity needs of breastfeeding support among participants [30]. Mothers were included if they (1) had infants with a gestational age of less than 37 weeks; (2) were aged between 20 and 50; (3) without psychiatric disorders. Mothers were excluded if they (1) had contraindications to breastfeeding such as active tuberculosis infection, HIV infection, receiving radioisotope diagnosis or treatment, and taking antimetabolic drugs or chemotherapy drugs; (2) had been artificially feeding their infants since birth.

### Data collection

The eligible mothers were identified on the day of discharge and informed them of the study via oral and written communications. The mothers who voluntarily participated in this study were required to sign a written informed consent and provide their personal WeChat (a popular communication application in China) number for keeping in touch after discharge. After days of breastfeeding at home, mothers were invited to a face-to-face semi-structured interview. Due to the COVID-19 pandemic, interviews were held online using Tencent Meeting software (a professional conference software in China like Zoom), with instant video and recording functions.

The interview guide was developed according to the BCW intervention guide, which had clearly listed exemplar interview questions [25]. Then it was piloted with two preterm infants’ mothers, without revisions needed (Additional file 1). The interviewers were all nursing graduate students who had received comprehensive training in qualitative research methodology and were experienced in conducting interviews. In preparation for the study, they were given interview guide to follow while also being allowed flexibility in the order of questioning and encouraged to ask probing or additional questions as interesting concepts emerged. Interview techniques, including silence, echo, verbal agreement, repetition, and ‘tell me more’ were used to gain further insight into mothers’ needs [31]. To avoid poor network connectivity, consensus was reached that participants should conduct a pretest of their internet connection. Within 24 h of completing each interview, the interviewer would transcribe the data into a written format and send it to each interviewee for review and confirmation. The interviewees were also given an opportunity to provide any additional information they felt was relevant. Data collection was undertaken from June to August 2022. Ten interviews were conducted before thematic saturation was reached, and three additional interviews were held to ensure that the information obtained was no longer new or surprising. The interviews lasted between 31 and 95 min, with a mean of 47 min, a median of 45 min, and were audio recorded. To ensure anonymity, all participants are represented by codes (M1-M13).

### Data analysis

All interview data were transcribed verbatim and managed using qualitative data analysis software NVivo 11 (QSR International). Then the audio recording was verified for transcription accuracy. Theoretical Thematic Analysis (TTA), a technique for extracting concepts and meanings from qualitative data and examining themes within a specific theory, was conducted to review session transcripts [30]. The recursive analysis phases included the following: (1) familiarization; (2) generating initial codes; (3) searching for themes; (4) reviewing themes; (5) defining and naming themes; and (6) producing the report.

During step 1 and 2, the audio recordings of interviews were reviewed repeatedly to ensure data familiarization. Following that, the COM-B domains (capability, opportunity, and motivation) of BCW provided a priori framework of themes for data analysis. Recurring statements related to them were generated as initial codes after line-by-line data analysis. During step 3, similar codes were categorized into subthemes, which were explained separately. And relevant subthemes were provisionally grouped into themes based on their shared characteristics. During step 4, the relationship between codes, subthemes, and levels of themes was discussed repeatedly until a consensus was reached. In the last 5 and 6 steps, themes were deductively mapped to the COM-B system, and were named with BCW terminology to identify maternal needs for enabling breastfeeding. Any disagreements were discussed and resolved in sessions twice weekly during data analysis.

## Results

The study included 13 mothers of preterm infants (median age 32 years, range 26–44 years). Ten mothers had cesarean deliveries, while three had spontaneous delivery. Most (n = 7) mothers were interviewed two weeks after discharge, while others (n = 3) were interviewed between three to four weeks and one to two months after discharge. Feeding modes of mothers included mixed breastfeeding (n = 7) and exclusive breastfeeding (n = 6). Table 1 lists the demographic characteristics of the participants.

**Table 1:** Participant characteristics (n = 13)

Characteristics	Frequency (n)
Age range	
20–30 years	6
31–40 years	5
>40 years	2
Gestational level	
<28 weeks	1
28–32 weeks	2
33–35 weeks	7
36–37 weeks	3
Length of separation	
<1 week	4
1–2 weeks	4
>2 weeks	5
Feeding mode	
Mix breastfeeding	7
Exclusive breastfeeding	6
Parity	
Unipara	9
Multipara	4
Delivery mode	
Spontaneous	3
Cesarean	10
Educational level	
Upper secondary school or less	4
Higher education	9
Employment status	
Unemployed	3
Maternal leave	10
Maternal leave	
158 days	5
173 days	4
188 days	1
Occupation	
None	3
Private enterprise	3
State enterprise	2
State institution	5
Monthly family income	
<6000 RMB	2
6000–11,999 RMB	7
>12,000 RMB	4

***RMB*** **Ren Min Bi**

Eleven subthemes were identified based on the data in the context during the analysis. Then, the findings were deductively mapped to higher-level domains of the BCW and lower-level domains of the TDF, resulting in five themes: (1) capability; (2) physical opportunity; (3) social opportunity; (4) reflective motivation; and (5) automatic motivation *(*Table 2*)*.

**Table 2:** Overview demands to enhance breastfeeding behaviour and their corresponding COM-B component and TDF domain

COM-B component	TDF domain	Subthemes	Demands description
Capability	Memory, attention and decision process	Understanding infants’ cues and identifying problems	Develop capability to read cues and identify abnormal infant behaviors so that can react promptly when challenges arise.
	Knowledge & skills	Breastfeeding knowledge accumulation and skills training	16 learning demands, of which the lactation education and breast pump selection guidance were the most frequently mentioned. Face-to-face, systematic, staffed and plain course. Educational resource that integrated text, image, and video modes.
Physical opportunity	Environmental context and resource	Cleanliness and quietness in the household environment	A clean and quiet household environment during postpartum recovery period.
		Breastfeeding support in workplaces Breastfeeding support in hospitals	Tools for breast milk storage, privacy lactation spaces, and flexible schedule for lactation in workplaces Private lactation spaces and tools for breast milk expression and storage in hospitals
Social opportunity	Social influences	Partner and family support	Family members share responsibility and have close collaboration to care for the infant.
		Peer support	Practical and emotional support provided by experienced mothers.
		Authoritative support from healthcare providers	Authoritative affirmation given by healthcare workers when women’s own breastfeeding advocacy was dismissed by family members.
Reflective motivation	Beliefs about consequences	Information about the health impacts of breastfeeding	Information on the health impacts of breastfeeding for mothers and infants, respectively.
Automatic motivation	Emotion	Maternal-infant bonding	Information about infant when separated by pictures, videos, phone call or internet.
		Free of aversive stimulus	No questioning and pressure but respect and comfort.

*COM-B* Capability, Opportunity, and Motivation System, *TDF* Theoretical Domains Framework

### Theme 1: capability

Capability refers to the relevant knowledge and skills to perform breastfeeding successfully.

### TDF domain: memory, attention and decision process

#### Understanding infants’ cues and identifying problems

Most mothers thought it was important to recognize when the baby is hungry or full in case of insufficient intake or unnecessary spitting up. Without the capability of reading infants’ cues, mothers were often uncertain while breastfeeding at home, which led to anxiety. Mothers stated that they desired a specific answer as to whether their breastfeeding was effective or not.

*“He is so little and weak. A normal baby can eat himself enough. However, my baby requires more time because I feel the suction is weak. I cannot tell if he is full or tired when he stops eating. It causes me great concern.” (M3, multipara, 34*^*0/7*^*weeks, 7 days of separation)*.

*“The nurse instructed me to feed her as soon as she became hungry, but how can I swiftly determine if she is hungry? I was uncertain what she was saying to me when crying. It may be that her diaper is full, she does not feel safe, or a startle reflex comes up. I need assistance interpreting my infant’s cues.” (M2, unipara, 33*^*0/7*^*weeks, 5 days of separation)*.

Moreover, they are eager to know whether their baby is continuing to be healthy after discharge. If the baby is becoming unwell, they desired to have the ability to recognize it as soon as possible.

*“I would like a list that specifies the normal amount of human milk and urine, and the normal appearance of human stool. It does not need to be precise, but at least let me know when to become alert.” (M11, multipara, 34*^*0/7*^*weeks, 14 days of separation)*.

*“When he sucks, his face easily turns red with body twisted, and he will spit up a little. I often worry that he is uncomfortable and become sick again.” (M9, unipara, 30*^*6/7*^*weeks, 43 days of separation)*.

In summary, most mothers wished to develop the ability to read infant cues and identify problems to breastfeed properly, confidently, and react promptly when problems arise.

### TDF domain: knowledge; skills

#### Breastfeeding knowledge accumulation and skills training

All the mothers encountered challenges in breastfeeding. Interviews unveiled 16 categories of knowledge and skills that mothers aspired to learn in order to cope with difficulties (Table 3*)*.

**Table 3:** Learning demand

Coping knowledge and skills	Participants
The meaning of breastfeeding	M1, M4-9, M11
The importance of colostrum	M2
The importance of early lactation	M1, M2, M5, M8
Lactation guidance	M1-3, M5-10
Selection of breast pump	M1-9, M11-12
Breastfeeding posture	M1, M3, M12
Breastfeeding technique for nipple attachment	M1, M3, M12
The collection, storage, transfer, thawing and heating of human milk	M6, M9
Use and precautions for human milk fortifier	M1, M2, M6
Nipple care	M1-3
Prevention and treatment of vomiting	M1, M3-4, M9, M11
Prevention and treatment of choking on milk	M1, M2-3, M9, M11
Observation of urine and stool	M2, M5, M7, M11
Observation and care of abdominal distension	M1, M5, M8, M11
Differences between preterm and full-term infants	M4, M8
Breastfeeding in particular situations (breast milk jaundice, cytomegalovirus infection, etc.)	M2, M3, M6, M9-10

The majority of mothers consistently perceived insufficient breast milk production, which in turn influencing their beliefs about their own breastfeeding capabilities. Thus, lactation guidance and breast pump selection were frequently cited as participants’ primary request.

*“I am nervous. My baby is so fragile, but I have insufficient breast milk (sigh). It would be great if there is any guidance to promote lactation.” (M3, multipara, 34*^*0/7*^*weeks, 7 days of separation)*.

*“I’m producing less and less breast milk these days. (…) While my baby was hospitalized, I had to rely on a breast pump. But the breast pump I randomly chose always caused discomfort and a tearing pain in my nipple when I used it. If only I had received some guidance on selecting a breast pump, I might have pumped more often.” (M6, unipara, 32*^*0/4*^*weeks, 14 days of separation)*.

Most mothers emphasized that face-to-face guidance before discharge could give instant feedback on whether their breastfeeding technique was correct. Some also believed that a standardized set of courses by a trained nurse was better than ad hoc instruction.

*“They can guide me (how to breastfeed) at discharge so that my wrong steps or postures can be corrected. If not, I have no idea whether I did it right or wrong when I returned home. It harms my baby and my breastfeeding confidence.” (M1, unipara, 35*^*0/7*^*weeks, 5 days of separation)*.

*“When I was hospitalized in the maternity ward, the nurse would not give formal and systematic training because she was too busy. She only gave guidance when she occasionally came in and felt that the new parents were clumsy. (…) Sometimes I wanted to ask for their instruction, but I was always unsure if it would interfere with their daily work. It would nice if there were a special nurse in charge of teaching.” (M11, multipara, 34*^*0/7*^*weeks, 14 days of separation)*.

Mothers also agreed that a straightforward and fun course was more accessible to as they lacked medical background.

*“I hope the class can be fun. Maybe you can teach us using models.” (M2, unipara, 33*^*0/7*^*weeks, 5 days of separation)*.

Most participants stated that there was too much information to grasp fully and worried they would forget or make mistakes, leading to bad outcomes. Thus, many wanted a database containing relevant information about knowledge or skills that could be referred to as they liked. Several mothers believed written materials with images could be effective, while others preferred video.

*“My husband was taught how to collect, store, and transfer human milk in the hospital, but he could not remember everything when he got home. Later, he took home written material and reviewed it periodically. It serves as a reminder and is convenient to annotate. (M8, unipara, 32*^*5/7*^*weeks, 35 days of separation)*

*“My time is fragmented as mother. Nowadays, short-form videos are very popular, and I think this will suit me well.” (M4, unipara, 35*^*4/7*^*weeks, 10 days of separation)*.

*“Some skills or techniques are more intuitive through video demonstrations, while text or pictures sometimes cause misunderstandings.” (M7, unipara, 28*^*2/7*^*weeks, 73 days of separation)*.

Overall, mothers were willing to learn relevant knowledge and skills, especially on lactation education and breast pump selection guidance. They hoped the course mode could be face-to-face, systematic, staffed and easy to understand. Additionally, they required an educational resource that integrated text, image, and video modes.

### Theme 2: physical opportunity

Physical opportunity refers to environment that makes breastfeeding physically accessible and affordable in terms of resources, locations, time and triggers [25].

### TDF domain: environmental context and resource

#### Cleanliness and quietness in the household environment

Keeping the home clean and hygienic was a need identified by some mothers because preterm infants were more susceptible to infection. Some participants expressed concerns regarding the potential for increased risk of infections and elevated noise levels due to visits from relatives who came to give their congratulations.

*“After the baby was discharged from the hospital, many elders came to visit and congratulate me at home, but each time I felt like I was facing the enemy. They hugged and kissed the baby. Even when I breastfed, female elders did not shy away. It was uncomfortable and stressful. (M2, unipara, 33*^*0/7*^*weeks, 5 days of separation)*

*“I have stringent rules at home. If someone wishes to touch my baby, he must wash his hands first and is not allowed to enter the baby’s room. But some people cannot understand. They will think, ‘Is it necessary to make such a fuss about caring for a preterm baby?’” (M5, multipara, 33*^*6/7*^*weeks, 46 days of separation)*.

In brief, mothers desired a clean and quiet home environment conductive to breastfeeding.

#### Breastfeeding support in workplaces

Women have multiple social roles and after their postpartum recovery they gradually returned to spaces outside of their home. Women wishing to continue breastfeeding hoped that society would strengthen their resolve by providing more breastfeeding resources to support their breastfeeding behavior.

*“I have some friends who actively and passively weaned their babies when they returned to work. (…) As far as I know, my workplace does not provide a refrigerator for human milk storage, let alone a lactation room.” (M8, unipara, 32*^*5/7*^*weeks, 35 days of separation)*.

*“My superior does not value breastfeeding. After all, there are not many people who have this demand, so I think it is unrealistic to expect them to provide a lactation room especially for us. But at least there should be a private area where I can pump my milk freely.” (M1, unipara, 35*^*0/7*^*weeks, 5 days of separation)*.

*“Once I return to work, time can be pretty tight. I need to wake up at 6am every day to catch the subway for work. (…) It just feels like juggling work and breastfeeding will be tough.” (M3, multipara, 34*^*0/7*^*weeks, 7 days of separation)*.

Overall, tools for breast milk storage, privacy spaces, and flexible schedule for lactation are what mothers need in workplaces.

#### Breastfeeding support in hospitals

After preterm infants were discharged from the hospital, they required regular follow-up, so the hospital environment and facilities were frequently mentioned as needing improvement.

*“Initially, I thought the nursing room might be similar to that in the mall, but later I noticed it was at the end of the aisle with a small table and sofa, even without a door. The hospital was extremely crowded. Despite my embarrassment, I had to breastfeed my hungry baby.” (M9, unipara, 30*^*6/7*^*weeks, 43 days of separation)*.

*“I had cytomegalovirus in my breast milk. When I brought my baby for follow-up, I have to take a bottle of frozen human milk with me for pasteurization. However, no milk heater was available. I had to breastfeed him directly. (…) Hospitals should be equipped with enough breastfeeding facilities and aids. For example, we need nursing rooms, changing tables, sofas, milk heaters, water dispensers, sinks, sanitizers, trash cans and refrigerators.” (M11, multipara, 34*^*0/7*^*weeks, 14 days of separation)*.

In short, they hoped there would be private spaces and tools for milk expression and storage in hospitals, which could make breastfeeding more convenient and dignified.

### Theme 3: social opportunity

Social opportunity refers to environment that makes breastfeeding socially acceptable, including interpersonal influences and cultural norms.

### TDF domain: social influences

#### Partner and family support

Participants shared a common problem: despite not fully recovering from the post-natal state, they had become the primary caregivers for their babies. Several mothers reported that they were not resting at home at all but working another way. The household chores and childcare tasks kept them busy and tired, distracting them from breastfeeding. Mothers agreed that breastfeeding was their own duty, but other baby care was the shared responsibility of the whole family.

*“Caring for her is not as simple as it seems because it takes me a lot of time and energy. (…) I gave birth and breastfed her, but caring for her is not solely my duty. I need assistance from my husband and my mother-in-law. (M2, unipara, 33*^*0/7*^*weeks, 5 days of separation)*

*“My husband offers me little help. Most of the time, he wants to help, but he does poorly. Luckily, my mother helps a lot. She can provide me with a nutritious diet and help with a diaper change, allowing me to focus on breastfeeding. However, sometimes views are diverse between old and new generations, and we will have disagreements in which neither of us can convince the other. (…) I hope all my family can be educated so there will be less disagreement and more integrated care.” (M11, multipara, 34*^*0/7*^*weeks, 14 days of separation)*.

In summary, mothers considered shared responsibility and close collaboration important for infant care in their family. They needed family support to have more energy and time to breastfeed.

#### Peer support

Peer support, along with family support, was a critical component of social resources. A sense of disconnection with medical staff or a lack of breastfeeding experience increased peer communication requirements.

*“ Communication between mothers is cordial. Perhaps similar people are more empathetic. (…) Medical staff may only communicate with you about the baby’s condition, but mothers can converse freely. Breastfeeding postures, recipes to promote lactation, and comfortable milk pumps are all meaningful topics.” (M2, unipara, 33*^*0/7*^*weeks, 5 days of separation)*.

*“I think the hospital can give chances for mothers to meet and interact so that we can learn and discuss with each other. (…) I am inexperienced, so if my baby behaves unusually, like a milk disc in the stool or refusing to eat, I will be worried about whether I should take him to the hospital or keep him under observation at home. However, if I know that other children have behaved like this before, and their mothers have feasible solutions, then my anxiety will dissipate.” (M7, unipara, 28*^*2/7*^*weeks, 73 days of separation)*.

In contrast, several mothers exhibited less need for peer support. One mother said:

*“I prefer quietness and I do not desire to talk to others. It is sufficient to have my family and doctors helping by my side.” (M5, multipara, 33*^*6/7*^*weeks, 46 days of separation)*.

Overall, mothers who lacked breastfeeding experience, family support, and professional support were more inclined to seek peer support.

#### Authoritative support from healthcare providers

Some mothers believed that in addition to educating mothers, a particular ‘function’ of healthcare workers was advocating for mothers when their voices were barely heard within their family.

*“Most people respect doctors naturally. When I complained of my depression and exhaustion, my family took me with a grain of salt, doubting if I was trying to be lazy. However, when doctors discussed postpartum depression and advocated for family corporation, they were more likely to believe and act.” (M2, unipara, 33*^*0/7*^*weeks, 5 days of separation)*.

*“What I said did not work. My mother tended to feed my baby water after each meal. I knew it was wrong but she insisted. The doctor was about to provide feeding instructions during a follow-up visit. Then I said, ‘hold on, please, I would like my mother to come in and learn together.’ (…) My mother never fed the baby extra water again after that. As a preterm mother, I hope the medical staff can instruct mothers and family members.” (M11, multipara, 34*^*0/7*^*weeks, 14 days of separation)*.

In brief, healthcare workers were expected to provide authoritative support when mothers were not listened to.

### Theme 4: reflective motivation

Reflective motivation refers to motivation that arises through reflection process, which involves plans (self-conscious intentions) and evaluations (beliefs about what is good and bad) [25].

### TDF domain: beliefs about consequences

#### Information about the health impacts of breastfeeding

Several primiparas recalled that their healthcare providers did not mention the need to pump breast milk early and regularly after delivery. They were initially unaware of the consequences of not doing it.

*“I did not realize the importance of lactation on time until cuirushi (unlicensed layperson who helps a mother lactation by massaged her breasts) in the confinement center instructed me to lactate every 3 hours. Why did no one inform me of this while I was hospitalized? I am now worried about insufficient lactation.” (M8, unipara, 32*^*5/7*^*weeks, 35 days of separation)*.

There was also a common perception among mothers that breastfeeding was better than formula feeding. Still, most of them did not know about the health impacts of breastfeeding, weakening their determination and motivation. Some mothers even doubted themselves, believed that formula was superior, and worried breastfeeding might lead to negative outcomes.

*“Almost everyone tells me that human milk is more nutritious, but I know that formula-fed infants grow faster than breastfed babies. The confusion discourages me and makes me want to quit.” (M9, unipara, 30*^*6/7*^*weeks, 43 days of separation)*.

Several mothers noted that babies should not be the only focus of breastfeeding, and that the benefits of breastfeeding for mothers should also be widely publicized.

*“Not all mothers can stick to breastfeeding because it may cause nipple pain, poor sleep quality, and being out of shape. You should put yourself into mothers’ shoes and make them realize that the benefits of breastfeeding do not lay only in babies.” (M2, unipara, 33*^*0/7*^*weeks, 5 days of separation)*.

In summary, information on the health impacts of breastfeeding was needed, and including why breastfeeding is better and how it impacts both mothers and infants, thereby strengthening their determination to continue breastfeeding.

### Theme 5: automatic motivation

Automatic motivation refers to motivation aroused by emotional reactions like desires, impulses, inhibitions and reflex responses [25].

### TDF domain: emotion

#### Maternal-infant bonding

Most mothers described their contact with babies as happiness, peace and harmony, making them feel like “moms”.

*“When I held him, even though he could not speak, his eyes gazed at me. It was like a secret spiritual communication, and I enjoyed the breastfeeding moment. (…) Sometimes they (babies) cried, and no one could successfully comfort them except me.” (M5, multipara, 33*^*6/7*^*weeks, 46 days of separation)*.

*“I remember the first time I breastfed, she lay on me, and it was a subtle, little cry-for-joy moment. I realized I had become a mother. Before that, I pumped my milk and sent it to NICU to find myself like a milk machine.” (M2, unipara, 33*^*0/7*^*weeks, 5 days of separation)*.

All participants discussed that they couldn’t forget how much they missed their babies when hospitalized. Different views emerged regarding how they wished medical staff to help support bonding with their babies. Some participants wanted to view photos and videos of their babies, and a few mothers wanted to come to the NICU and stay together with their infants.

*“I missed him extremely. I wanted him to return soon! (…) You can send us videos or photos or something similar.” (M1, unipara, 35*^*0/7*^*weeks, 5 days of separation)*.

*“The confinement center I lived hired pediatric professors from hospitals for weekly rounds. I wondered why the hospital did not have its own parent-child unit in the NICU, where the baby can receive treatment while staying with me, and it’s also more convenient for breastfeeding.” (M5, multipara, 33*^*6/7*^*weeks, 46 days of separation)*.

In contrast, others believed that the scene of a baby living in an incubator with injections and probably tubes would increase their anxiety and heartbreak. Thus, they proposed a compromise:

*“I will be satisfied if I can get the most fundamental information about him, such as milk volume, temperature, and weight. A one-minute phone call after rounds could put my mind at ease.” (M3, multipara, 34*^*0/7*^*weeks, 7 days of separation)*.

*“The baby’s daily treatment information or progress could be posted on the hospital’s website so that the doctors and nurses do not have to spend time on feedback and we can access it ourselves. additionally, the written records can be checked repeatedly in case of noise or misunderstanding on the phone.” (M10, unipara, 34*^*2/7*^*weeks, 11 days of separation)*.

In short, most participants expressed that mother-infant contact and bonding could motivate breastfeeding. Mothers were in great need of information about their infants when they were apart. They felt that sending photos or videos was a straightforward way to provide information, while providing basic information via phone call or the internet was also important.

#### Free of aversive stimulus

A common sentiment among mothers was that they were not as valued as they were prior to the birth, with the baby receiving most of the family’s attention. It led to an awkward situation where some family members pressured mothers to eat and breastfeed for the baby’s benefit. This was uncomfortable and upsetting to some mothers.

*“My mother-in-law gives me plenty of pressure. Initially, I planned to breastfeed, but after my babies returned home from NICU, I have to take care of two children. I felt tired and had inadequate breast milk. She ignored the fact and continued to emphasize the importance of breastfeeding. It made me nervous, upset, and resist breastfeeding for a while.” (M5, multipara, 33*^*6/7*^*weeks, 46 days of separation)*.

*“(…) I once cried in front of my husband. I could not control my emotions. I felt like my husband no longer respected and paid attention to me. He only cared about whether or not the baby was full. The only attention I got was, ‘why do you lactate less? Why do you eat less? The baby will be malnourished.’ This makes me feel like I am no mother but a cow.” (M2, unipara, 33*^*0/7*^*weeks, 5 days of separation)*.

Overall, aversive stimuli could suppress mothers’ motivation to breastfeed. Mothers needed respect and comfort when faced with difficulties rather than questioning and pushing.

## Discussion

As far as we know, this is the first study utilizing the BCW to provide a comprehensive and overview of Chinese preterm mothers’ requests for how to better support breastfeeding. Collectively, five themes were identified based on the theoretical framework from semi-structured interviews: (1) capability; (2) physical opportunity; (3) social opportunity; (4) reflective motivation; (5) automatic motivation. Our results indicated that women’s needs were diverse regarding breastfeeding. Not only did they want to improve their breastfeeding knowledge and skills, but they also needed adequate support and external resources to be able to breastfeed, thus stimulating and maintaining their motivation for breastfeeding.

As regard to capability, our data reflected that mothers desired to improve their breastfeeding knowledge and skills, especially on lactation guidance for best practice, which was in line with previous studies on full-term infants [32]. However, there were also differences. One was that preterm mothers were also eager to receive guidance on pump selection. In China, preterm infants are usually admitted to the NICU after birth where parents cannot have contact with their infants [14]. This mother-infant separation deprives them of the opportunity to breastfeed directly, so they must rely on the breast pump to express milk and transfer breast milk to the NICU. Therefore, using the pump is essential for stimulating and maintaining lactation. The other difference was that preterm infants’ mothers attached great importance to improving their capability of recognizing infants’ cues and any concerning behavior, which was consistent with previous studies [19, 33]. The mother’s missed opportunity in the NICU to observe the infant’s behavior and they had difficulty recognizing their fullness and hunger cues after discharge because of their preterm infant’s weak suck and because they got easily fatigued from breastfeeding. Research has shown that mothers’ perception of breast milk supply also bases on their babies’ behaviour, particularly crying and fussiness [10] At the same time, preterm infants have a higher risk of feeding intolerance and NEC than full-term [34]. Hence, mothers needed training or guidance that enabled them to interpret their infants’ cues, and make competent decisions when problems arose.

As for the physical opportunity of breastfeeding, participants mentioned that current environmental resources in both homes and public places did not meet their needs. Previous studies primarily reflected mothers’ requirements for public places, whereas the need for a household environment was rarely reported [35, 36]. In this study, we revealed that mothers needed the home to be a clean and quiet place to breastfeed. In China, it is customary for friends and relatives to visit and congratulate the whole family at home after childbirth to share good luck and good omens. However, since preterm infants are vulnerable and poor in immune function, it becomes a “happy burden” for some mothers. They were aware that the visits were well-intentioned and a source of blessings, yet they struggled with managing the potential risk of infections and the stress on breastfeeding caused be these visits. In fact, the visits were not the main concern for mothers, but rather the thoughtless behavior of visitors that disrupted home environment. This dilemma was similar to the situation in most Chinese NICUs, and many of them chose to implemented a separation policy to ensure a clean and quiet medical environment [14, 37]. Just as the separation policy can have detrimental effects on the well-being of both mothers and babies, the refusal to social obligations to host visitors may adversely impact the familial social relationships. Perhaps only when parents were taught on improving their hand hygiene and infection prevention skills, will they be able to confidently educate visitors in turn, with the ultimate goal of protecting the cleanliness of the home environment.

Breastfeeding doesn’t always happen at home and babies can get hungry wherever they are. Similar to previous studies [38, 39], we found that breastfeeding facilities in public are still inadequate and cannot provide mothers with enough privacy and convenience, despite China having already issued national guidelines for it in 2016 [40].

The length and benefits of Chinese statutory maternity leave are among the highest in the world [41]. Women are entitled to 98 days of paid maternity leave plus extended maternity leave ranging from 30 to 267 days, as well as breastfeeding breaks ranging from 1 to 2 h per day for the first year after delivery [42]. These provisions align with or surpass the recommendations of the International Labour Organization (ILO) [43]. Thus, the salient need of women was not about maternity leave, but rather the availability of material resources within the workplace, such as private space, time, and refrigeration facilities for breastfeeding, which was similar to previous studies [42, 44]. In response to these needs, evidence has already showed that providing spaces, implementing flexible working arrangements for women (e.g., telecommuting), and providing breastfeeding tools can effectively support breastfeeding practices in the workplaces [45]. The implementation and maintenance of these measures, however, entail additional costs that are currently borne by the enterprises themselves in China [46]. Moreover, the provision of these material resources in workplaces is only the responsibility of the enterprise and not a legal obligation [46]. Therefore, if reasonable sharing of costs can’t be reached, businesses may be less motivated to implement breastfeeding policies and women may encounter difficulties in accessing the breastfeeding support they need within their workplaces.

The hospital environment also plays an important role as primary setting for delivery, breastfeeding, and health care for preterm infants. Some countries have implemented the family-integrated care in NICUs, wherein families are afforded family rooms equipped with amenities such as a refrigerator, air-conditioning, a bed (or sofa), and breastfeeding tools [47]. The care model provides opportunities for maternal-infant interaction and skin-to-skin contact during hospitalization, which can lead to better understanding of infant cues, maintenance of lactation, and increased motivation to breastfeed [47]. This is also recommended by WHO to implement in health-care facilities at all levels [15].

Considering social opportunities, family members, experienced mothers, and healthcare professionals are three primary groups of people that could provide mothers with more breastfeeding opportunities. Previous studies differed on whether families acted as facilitators or barriers to exclusive breastfeeding practices [48, 49]. Our study aligned with the newly released guidelines from the WHO for preterm infants [15], indicating family involvement can provide emotional support and alleviate parenting tasks and housework burden. Affected by the traditional gender roles that “the man goes out to work while the woman looks after the house”, women in China are expected to devote more to family labor than men [50]. However, dysphagia and weaker suck of preterm infants prolong breastfeeding time [11], leaving mothers even busier. Therefore, it is unsurprising that mothers of preterm babies expressed that they needed family members to share household and parenting tasks to enable more breastfeeding opportunities. According to the WHO’s guideline [15], emotional support from other parents in similar situations is also valued by many mothers. To maintain breastfeeding, a sense of connection and a desire for a group that shares similar goals and challenges is needed. Although peer support may not provide breastfeeding health information that can be obtained from a healthcare provider, it is a more accessible resource with fewer limits for preterm mothers. Healthcare providers also play a key role in social opportunities. In contrast to previous studies that highlighted the need only for professional guidance provided to mothers themselves [32, 50], this study emphasized the need for healthcare providers to serve as an authority to speak on behalf of mothers to their family members to reinforce the importance of breastfeeding. In Chinese culture, once a woman marries, she no longer belongs to her birth family but instead joins her husband’s family and becomes a new member of that family [51]. Mothers often lack authority in this new environment and seldom receive the attention or support they need when disagreements arise. This could explain the need for outside authoritative support from healthcare providers.

With reference to reflective motivation, there was a great need for information on the health impacts of breastfeeding. As with previous studies, the health benefits of breastfeeding are potent motivating factors for implementing breastfeeding behaviors [12, 52]. Current breastfeeding promotion practices emphasize the nutritional benefits to the infant [23], which is important, but should not be the sole concern. If individuals do not believe they will benefit from the behavior, then they are more likely to abandon it. In addition, many mothers had misconceptions about breastfeeding that it could lead to nipple soreness and a loss of shape, reflecting a lack of information and knowledge support. Identifying and promptly correcting common misconceptions, and increasing women’s awareness of the wide benefits and joys of breastfeeding, could potentially serve as a strategy for healthcare providers to enhance women’s motivation.

As regards automatic motivation, mothers’ intrinsic motivation to breastfeed required more positive emotional responses. According to a previous study, maternal-infant separation can result in numerous psychological problems for mothers, including stress, anxiety, depressive symptoms, and feelings of being unable to care for their babies [37], which may hinder mothers’ emotional motivation to express breast milk [53]. Moreover, it is strongly recommended by WHO that KMC and breastfeeding be initiated as early as possible for all preterm and low-birth-weight infants [15], which will be difficult to achieve if mother and baby are separated. Therefore, it is crucial to abandon the outdated separation policy so that mothers and babies can stay together.

Additionally, we found that certain aversive stimuli, including coercion and objectification of breastfeeding must be eliminated because they made mothers feel worse emotionally and reduced their motivation to breastfeed. Mothers of preterm infants encounter more challenges and suffer from more aversive stimuli during breastfeeding. However, the majority of mothers in our studies are the post-1980s and post-1990s, belonging to the Chinese “one-child” generation released in 1979, who are better educated, bolder, with higher self-expectations and more robust sense of independence [54]. Therefore, when aversive stimulus contradicts women’s self-esteem and affects their emotions, they are brave enough to reveal it.

Notably, the needs of preterm mothers investigated in this study cannot be achieved by the mothers alone. Future research should focus on how to actively engage people, resources, and environments associated with the needs so that they can help enhance capability, create opportunities, and promote motivation to support breastfeeding behaviors. For example, firstly, proper consideration should be given to the policy of “zero separation” between families and their preterm infants. Separation can lead to maternal psychological problems and reduced lactation [37]. It also poses significant barrier to the initiation of early KMC and skin-to-skin contact, both of which are crucial interventions that lay the foundation for successful breastfeeding outcomes and are associated with reduced mortality and morbidity rates in preterm infants [15]. Secondly, inequities in breastfeeding should be taken seriously, and practical solutions should be found. Research suggests that women with higher socioeconomic status are more likely to breastfeed than those of lower status [55]. To motivate them intrinsically, more attention should be paid to women’s health, lives, and experiences, and psychological interventions should be implemented to stimulate positive emotions and thoughts [56]. The benefits of breastfeeding for mothers and infants and the labor required should be widely publicized through high-level advocacy, news reports, or public service announcements, making breastfeeding a socially valued norm.

The results of our study add valuable information to the global literature on breastfeeding. Firstly, the needs of mothers of preterm infants are diverse, and unique due to Chinese breastfeeding culture and the postnatal maternal-infant separation policy in most NICUs. Secondly, our qualitative research was guided by a comprehensive theoretical framework, which make it more rigorous. Lastly, to the best of our knowledge, the BCW is used for the first time to gain insight into women’s needs for implementing breastfeeding behavior. Adaptation of this research in future intervention design may offer reference to enhance the breastfeeding behavior of preterm infants’ mothers with similar backgrounds.

### Limitations

We recognized that there are several limitations. First, participants were recruited exclusively from a tertiary hospital in an urban area. Thus, findings in this study need to be ascertained by future investigations that include mothers from broader sociodemographic samples. Second, women’s needs for breastfeeding and enhancing the maternal-infant bond during hospitalization were reported after discharge, which may lead to recall bias. In future research, interviews could be conducted during the hospitalized period. Lastly, formula feeding, as a competing behavior to breastfeeding, is less explored in this study. Therefore, future studies could explore this more comprehensively to facilitate breastfeeding behavior and promote maternal-infant health.

## Conclusion

In this study, we firstly used the BCW to identify what mothers of preterm infants in China believe would enable them to breastfeed. We found that mothers needed to increase their breastfeeding capability, physical and social opportunities, and reflective and automatic motivation. To meet their needs, it is imperative to implement “zero separation” policy in the NICU, along with early KMC and skin-to-skin care. People, resources, and environments associated with these needs should also be engaged together to stablish a conducive structural environment for breastfeeding. Our study adds valuable information to the global literature on breastfeeding. Future studies are needed to design and implement effective interventions according to the specific needs of mothers to enhance breastfeeding behavior.

### Electronic supplementary material

Below is the link to the electronic supplementary material.

Supplementary Material 1

## Data Availability

The datasets used and analysed during the current study are available from the corresponding author on reasonable request.

## Abbreviations

ROP: Retinopathy of Prematurity
NEC: Necrotizing Enterocolitis
WHO: World Health Organization
KMC: Kangaroo Mother Care
BCW: Behaviour Change Wheel
COM-B: Capability, Opportunity, and Motivation System
TDF: Theoretical Domains Framework
COREQ: Consolidated Criteria for Reporting Qualitative Research
TTA: Theoretical Thematic Analysis
ILO: International Labour Organization
