# Supplementary material for: Breastfeeding needs of mothers of preterm infants in China: a qualitative study informed by the behaviour change wheel
Source: Int Breastfeed J. 2023 Sep 1;18:50. doi: 10.1186/s13006-023-00587-9 (PMC10472562; doi:10.1186/s13006-023-00587-9)
Supplement: Supplementary file 1 — Supplementary Material 1 [file 13006_2023_587_MOESM1_ESM.doc]

**Additional file 1: Interview guide**

| Introduction | | |
| --- | --- | --- |
| Hello, recently we have been designing a breastfeeding training project for mothers of preterm infants. In order to enhancing breastfeeding behaviors, we would like to have an in-depth interview with you to discuss what difficulties you encounter and what you need as a preterm mother during your breastfeeding. We hope you can provide us with as much information as possible. Your efforts will undoubtedly contribute to our project, which in turn will benefit more mothers and preterm babies. The interview is needed to be recorded later. Your personal information will be kept confidential and used only for scientific research. Ethics approval has been obtained from the Medical Ethics Committee of Tongji Hospital so that it won’t harm you or your baby. That’s all about this interview you may want to know. So if you are ready for this, let me ask you for some basic information… (age, maternal working status, education level, delivery mode, parity, feeding mode, discharge days, gestational age, birth weight) | | |
| COM-B | TDF | Interview Questions |
| Capability | Knowledge | -Do you know how to breastfeed your preterm baby? (Prompt-Could you please describe the feeding procedure?)  -Do you have any confusion or demands during the procedure of breastfeeding? (Prompt-What would you like us to teach you about the knowledge of breastfeeding? |
|  | Skills | -What would you like us to teach you about the skills of breastfeeding? |
|  | Memory, attention and decision Process | -How do you ensure you don't forget the breastfeeding knowledge or skills you previously learned? (Prompt-What do you need to help you gradually master breastfeeding knowledge and skills?) |
| Opportunity | Social influences | -How does your family influence your feeding mode?  -How do other mothers of preterm infants influence your feeding mode?  -How medical staffs influence your feeding mode?  -What help or support do you need from them? |
|  | Environmental context and resources | -What kind of environmental support or physical conditions do you need? |
| Motivation | Beliefs about consequences | -What factors influence your wish to breastfeed? (Prompt-family/social/one’s expectations; effects on maternal and infant health; consequences of not breastfeeding) |
|  | Emotion | -How does breastfeeding make you feel? (Prompt-What emotional responses have you experienced in breastfeeding?)  -Are there difficulties or problems that make you feel uncomfortable?  -How do your feelings affect whether or not you breastfeed?  -What do you think is needed to make you more comfortable/happy to breastfeed? |
| COM-B Capability, Opportunity, and Motivation System, TDF Theoretical Domains Framework | | |
